# Anaemia among Kenyan children: a call for improved monitoring and intervention in school-aged children

**DOI:** 10.1093/trstmh/traa032

**Published:** 2020-06-02

**Authors:** Emelda A Okiro, Noel K Joseph, Caroline W Gitonga, Robert W Snow

**Affiliations:** Population Health Unit, Kenya Medical Research Institute-Wellcome Trust Research Programme, PO Box 43640–00100, Nairobi, Kenya; Centre for Tropical Medicine and Global Health, Nuffield Department of Clinical Medicine, University of Oxford, OX3 7LG, Oxford, UK; Population Health Unit, Kenya Medical Research Institute-Wellcome Trust Research Programme, PO Box 43640–00100, Nairobi, Kenya; Population Health Unit, Kenya Medical Research Institute-Wellcome Trust Research Programme, PO Box 43640–00100, Nairobi, Kenya; Population Health Unit, Kenya Medical Research Institute-Wellcome Trust Research Programme, PO Box 43640–00100, Nairobi, Kenya; Centre for Tropical Medicine and Global Health, Nuffield Department of Clinical Medicine, University of Oxford, OX3 7LG, Oxford, UK

**Keywords:** anaemia, Kenya, prevalence, school-aged children

## Abstract

**Background:**

Anaemia has long been recognised as a major public health problem among young children in lower- and middle-income countries and is an indicator of both poor nutrition and health status. There has been little progress towards improvement of anaemia in part due to its complex aetiology. An added impediment to the progress is that the monitoring of anaemia does not routinely target the whole population, with school-aged children (SAC) largely overlooked.

**Methods:**

We re-examined data on the prevalence of anaemia among children aged <15 y sampled from 2008–2015 in Kenya.

**Results:**

Approximately one in four Kenyan children aged <15 y were described as anaemic, including 12% with WHO-defined moderate anaemia and 1% who were severely anaemic. Average haemoglobin concentrations increased with age and the risk of having anaemia decreased with age. However, one in five SAC in Kenya were suffering from anaemia; most were either mild (11.4%) or moderately (10.9%) anaemic.

**Conclusions:**

The monitoring of anaemia in SAC continues to be a neglected area limiting a careful articulation of the need to target interventions in this age group.

## Introduction

Anaemia remains a problem of global importance. It is a haematological state with a large consequential morbidity, mortality and delayed physical and cognitive development.^1^ While anaemia has been recognised as a concern for many years, there has been limited progress towards reducing the global burden posed by anaemia.^2^ In 2010, anaemia affected one third of the world's population and 50% of those living in sub-Saharan Africa (SSA).^2^ The intractability of the anaemia burden worldwide^3^ is, in part, a result of its complex, geographic-specific, multifactorial aetiology,^4^ requiring multiple integrated health interventions targeted at different age groups in each context.

To target interventions aimed at reducing anaemia demands an understanding of its variation by age, gender, pregnancy status, genetic, nutritional and environmental factors. However, most national nutrition, health and demographic surveys typically only sample children aged <5 y and women of reproductive age, hence much less is known about anaemia in other groups, especially preadolescent (aged 8–12 y) school-aged children (SAC).^5,6^ Here, we examine the prevalence of anaemia from school-based and household surveys among children up to their 15th birthday sampled from 2008–2015 in Kenya.

## Methods 

The data for this study were collected as part of school surveys undertaken from 2008–2010 during a collaboration between the Ministry of Education, Ministry of Public Health & Sanitation, World Bank and Kenya Medical Research Institute-Wellcome Trust^7^; and national household surveys that atypically sampled children up their 15th birthday, in 2010 and 2015, as part of the Kenyan Malaria Indicator Surveys (MISs). National school surveys and the two MISs were stratified based on five malaria risk zones and sampled to have adequate power at regional (province) and malaria ecological zone. Full sampling, methodological and ethical procedures are provided elsewhere for the MIS^8,9^ and the school surveys.^7^ In brief, alongside detailed health and intervention use questions, consenting participants in all surveys provided a finger- or heel-prick blood sample for haemoglobin (Hb) concentration that was measured in the field using a portable photometer (HemoCue AB, Ängelholm, Sweden). Hb measurements were adjusted for altitude as per Centers for Disease Control and Prevention recommendations (Hb = −0.32 × [altitude in metres × 0.0033] + 0.22 × [altitude in metres × 0.0033]). No adjustment is made for altitudes below 1000 m. Anaemia was defined using the following age-specific thresholds: <11 g/dl for children aged <5 y, <11.5 g/dl for children aged 5–11 y and <12 g/dl for children aged 12–14 y.^4^ Standard definitions of anaemia severity were defined, as shown in Table 1.

**Table 1.** Summary statistics of study population in 59 332 children aged 6 mo to 14 y in Kenya from 2008–2015

Characteristic		School survey 2008–2010, n (%)	MIS 2010, n (%)	MIS 2015, n (%)
Number of children		38 630	10 651	10 051
Number of clusters/schools		399	240	245
Gender	Female	19 281 (49.9%)	5340 (50.8%)	5086 (49.2%)
Age, y (median)		11 IQR (9–12)	6 IQR (3–10)	7 IQR (4–10)
Anaemia status*	Anaemic	9124 (23.6%)	3613 (32.5%)	2625 (25.1%)
	Mild anaemia	4673 (12.1%)	1605 (14.5%)	1313 (12.8)
	Moderate anaemia	4145 (10.7%)	1843 (16.8%)	1244 (11.6%)
	Severe anaemia	306 (0.8%)	165 (1.3%)	68 (0.6%)

Abbreviation: MIS, malaria indicator survey.

*WHO Hb age-specific cutoffs for anaemia were used: mild anaemia (defined as Hb <11 g/dl in children aged <5 y and Hb <11.5 g/dl in children aged 5–11 y and Hb <12 g/dl in children aged 12–14 y); moderate anaemia (defined as Hb <10 g/dl in children aged <5 y and Hb <11 g/dl in all other age groups); severe anaemia (defined as Hb <7 g/dl in children aged <5 y and Hb <8 g/dl in all other age groups.

## Results

Data were available for 59 332 children aged 6 mo to 14 y from the three surveys, consisting of 29 707 girls and 29 625 boys. They were sampled from 2008–2010 during the school surveys (38 630 children aged 4–14 y) and during 2010 and 2015 as part of the MISs (20 702 children aged 6 mo to 14 y) (Table 1). The mean prevalence of any anaemia was 25%, while that of mild, moderate and severe anaemia across all surveys was 13%, 12% and 1%, respectively. The prevalence of anaemia was 24% in the school survey (2008–2010) and 33% and 25% in the Kenyan MIS 2010 and 2015, respectively (Table 1).

**Figure 1.**
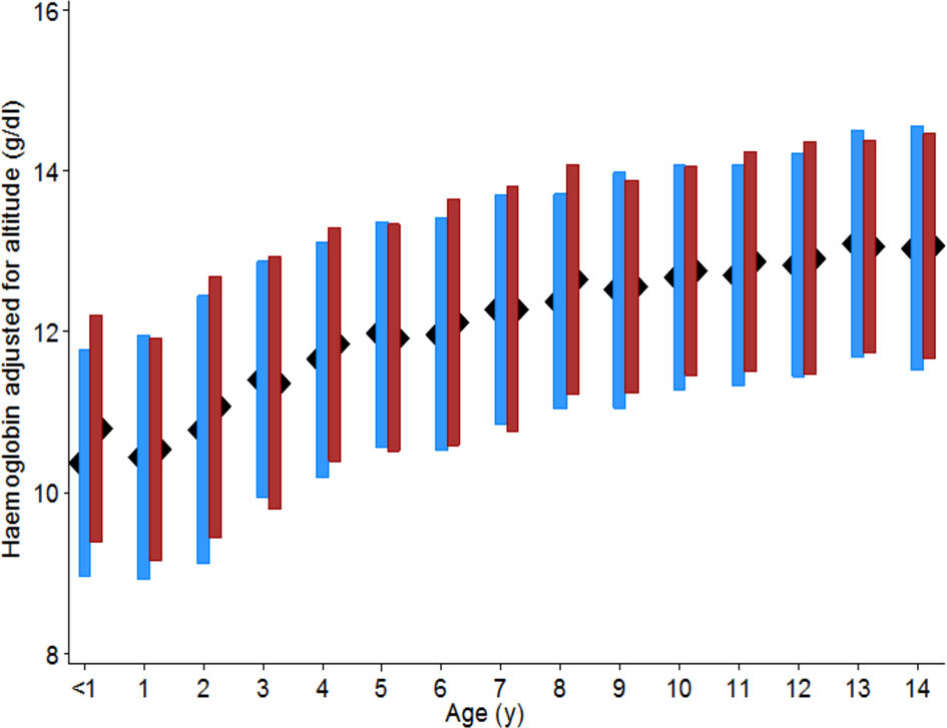
Trend of haemoglobin values with age and gender among children aged 6 mo to 14 y in Kenya. Averages and standard deviations are shown with males in blue and females in maroon. Children aged <1 y are 6–11 mo.

**Figure 2.**
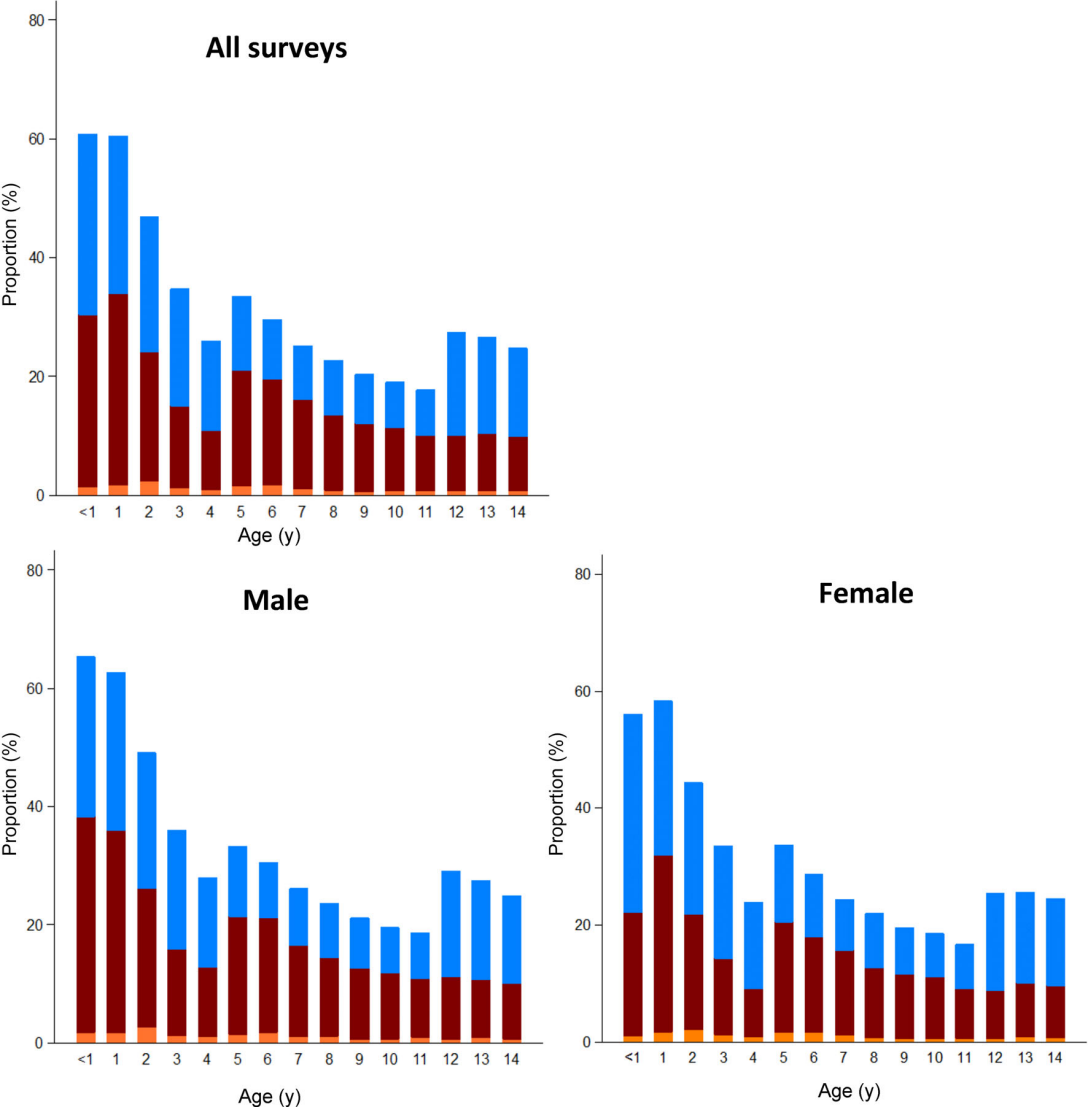
Severity of anaemia by age and gender among children aged 6 mo to 14 y in Kenya (definitions in footnote to Table 1). Children aged <1 are 6–11 mo.

Across all surveys, Hb concentration steadily increased with age (Figure 1). Mean Hb concentration was lower in males (12.3 g/dl) than females (12.4 g/dl) (p < 0.0001), a consistent finding across most age groups until around the age of 9 y (Figure 1). However, the prevalence of anaemia was highest in children aged 6 mo to 2 y and decreased with increasing age until 4 y. Also, 23% of SAC (5–14 y) were defined as anaemic, with a higher prevalence in males (22.3%) vs females (20.9%) (p < 0.0001) in this age group. The prevalence of mild, moderate and severe anaemia by age is presented in Figure 2. The highest prevalence of severe anaemia was seen in children aged 1–2 y (1.9%) and this pattern was the same in both males (2.2%) and females (1.5%) (Figure 2). The prevalence of anaemia in males was highest in infancy in the age group of 6–11 mo while in females the prevalence of anaemia was highest in children aged 12-23 mo. The prevalence of mild anaemia was observed to increase in children aged 12–14 y and this was observed in both males and females (Figure 2).

There was a significant difference in the proportion of moderate to severe anaemia between children aged 6 mo to 4 y (20.7%) compared with those aged 5 y and older (12.3%) (p < 0.0001). The proportion of children with mild anaemia was similarly high among children aged 6 mo to 4 y (21.6%) compared with those who were aged 5 y and older (11.4%) and this difference was significant (p < 0.0001).

## Discussion

From 2008–2015, approximately one in four Kenyan children aged <15 y were observed as anaemic, including 12% with WHO-defined moderate anaemia (Table 1). A point prevalence of 1% for severe anaemia recorded among school attendees or those residing in the household suggests that these children may develop clinical states demanding blood transfusion services or remain under the radar of emergency interventions, both carrying an increased risk of death.

Average Hb concentration increased with age, while the risk of having anaemia decreased with age (Figures 1 and 2). This is characteristic of many low and middle-income country contexts, where early childhood is a time of higher risks posed by micronutrient-deficient diets and the impact of acute infectious diseases. Targeting young children as part of nutritional and disease prevention strategies remains central to attempts to reduce the national burden of anaemia. However, the analysis also demonstrates significant levels of anaemia among SAC (aged 5–14 y) (Table 1; Figures 1 and 2). Approximately one in five SAC in Kenya were suffering from anaemia; most were either mild (11.4%) or moderately (10.9%) anaemic while a minority (0.8%) were severely anaemic. Considering the assumption of national representativeness of the survey data and national estimates of primary school enrolment in Kenya in 2012 (https://data.worldbank.org/country/kenya), approximately 1.5 million children attending school are anaemic and 0.7 million children have moderate anaemia. These results highlight important insights regarding the magnitude of anaemia as a public health problem extending beyond younger children to SAC. This is a subset population where the existing knowledge regarding the prevalence of anaemia is grossly inadequate.

Anaemia in this age group has been associated with poor cognitive development^1^ and arises through combinations of factors including repeated infections or chronic malaria and helminth infections^10^ and the rapid physical and physiological development associated with this age group. Additionally, a large proportion of children in SSA carry the sickle cell trait, a long-term protective consequence of living in historically malaria-endemic areas. A consequence of improved child survival of homozygotes (SS genotype) also means that these children will reach school age but remain in stable, anaemic states.^11^ SAC should not be neglected as targets for interventions aimed at reducing the anaemia burden. They remain at risk and can be targeted with specific interventions aimed at reducing parasitic infections, as well as supportive iron supplementation and the practical combination of interventions within the school environment.^12^

There are varying reports in the literature regarding the prevalence of anaemia in boys compared with girls.^13^ Data from Kenya suggest that mean Hb was slightly lower and that there were marginally higher risks of anaemia in males across most age groups before the age of 10 y. These findings were not significant but are worthy of further exploration. This has been reported previously in Benin and Mali.^6^

Without empirical data, modelling national burdens of anaemia beyond early childhood to older age groups may be accompanied by extrapolation errors. For example, the study by Kassebaum et al.^2^ suggests that the prevalence of anaemia in Kenyan children aged 5–14 y was 55%, 27% higher than that documented in the national school and household surveys. Anaemia is a valuable biomarker of broad population health status and has been used as a marker of disease-specific intervention success including helminth control^14^ and is increasingly promoted as a marker of malaria control progress.^15^ However, anaemia has not been reported consistently as an indicator of measuring progress or used for tracking health status and well-being; the most recent estimates reported by WHO are from 2011. These estimates also do not routinely sample whole populations and are similarly deficient in estimates for specific age-groups, nor do they discriminate by gender, both of which are important sources of heterogeneity.

## Conclusion

Anaemia is a recognised major public health problem among infants in developing countries. The monitoring of anaemia in SAC continues to be a neglected area,^5^ limiting a careful articulation of the need to target interventions in this age group.^16^

## Data Availability

The MIS datasets analysed during the current study are available through the Demographic and Health Surveys data portal, https://dhsprogram.com/data/available-datasets.cfm, upon request. The school survey datasets analysed during the current study are available from the corresponding author upon reasonable request.
